# Recovery of cardiac electrophysiological alterations by heart rate complexity based on multiscale entropy following liver transplantation

**DOI:** 10.1038/s41598-024-58191-8

**Published:** 2024-03-29

**Authors:** Po-Yuan Shih, Ya-Jung Cheng, Shih-I Ho, Hui-Hsun Huang, Jia-Rong Yeh, Wei-Zen Sun, Kuang-Cheng Chan

**Affiliations:** 1 Department of Anesthesiology, National Taiwan University Hospital, No.7, Zhongshan S. Rd., Zhongzheng Dist., Taipei City 100, Taiwan; 2 Research Center for Adaptive Data Analysis and Center for Dynamical Biomarkers and Translational Medicine, National Central University, No. 200, Zhongbei Rd., Zhongli Dist., Taoyuan City, 320314 Taiwan

**Keywords:** Heart rate complexity, Heart rate multiscale entropy, Heart rate variability, Model for end-stage liver disease, Liver transplantation, Liver diseases, Diagnostic markers, Outcomes research, Translational research

## Abstract

Autonomic nervous dysfunction is a known cardiac sequalae in patients with end-stage liver disease and is associated with a poor prognosis. Heart rate analysis using nonlinear models such as multiscale entropy (MSE) or complexity may identify marked changes in these patients where conventional heart rate variability (HRV) measurements do not. To investigate the application of heart rate complexity (HRC) based on MSE in liver transplantation settings. Thirty adult recipients of elective living donor liver transplantation were enrolled. HRV parameters using conventional HRV analysis and HRC analysis were obtained at the following time points: (1) 1 day before surgery, (2) postoperative day (POD) 7, (3) POD 14, (4) POD 90, and (5) POD 180. Preoperatively, patients with MELD score ≥ 25 had significantly lower HRC compared to patients with lower MELD scores. This difference in HRC disappeared by POD 7 following liver transplantation and subsequent analyses at POD 90 and 180 continued to show no significant difference. Our results indicated a significant negative correlation between HRC based on MSE analysis and liver disease severity preoperatively, which may be more sensitive than conventional linear HRV analysis. HRC in patients with MELD score ≧ 25 improved over time and became comparable to those with MELD < 25 as early as in 7 days.

## Introduction

Cirrhotic cardiomyopathy is a clinical condition in liver cirrhosis that consists of systolic incompetence under the condition of stress, diastolic dysfunction related to altered diastolic relaxation, and electrophysiological abnormalities in the absence of any known underlying cardiac disease^1,2^. In addition, autonomic nervous dysfunction can also occur in in patients with liver disease and it can further contribute to cardiac and vascular dysfunctions in the setting of liver cirrhosis^3^.

Heart rate variability (HRV), which is a noninvasive measurement method based on electrocardiographic data, is useful for evaluating autonomic nervous system function^4^. Conventional HRV analyses (including frequency and time domain analyses) have been reported to have prognostic value for congestive heart failure^5^. Studies have also used HRV to evaluate the heart function of patients with liver cirrhosis, and they have reported that the degree of decreased HRV is related to the severity of liver disease^6–9^. In recent years, studies have recognized heart rate fluctuations as complex behaviors that originate from nonlinear processes, and these fluctuations often exhibit nonstationary property^10–13^. Sample entropy (SamEn) is used to estimate the irregularity of a nonlinear and non-stationary time series (e.g., heartbeat time series)^10^. Costa et al.^11^ proposed one of the nonlinear models, the multiscale entropy (MSE) that calculates entropy over multiple time scales^11^. In MSE, a coarse-graining process is used to merge non-overlapped data segments with a fixed length into new samples by averaging, and the length of data segment is defined as the scale denoted by *n*. A coarse-grained time series with scale of n has a data length 1/n time of the length of the original time series. The entropies of the coarse-grained time series can be calculated by the algorithm of SamEn to represent the irregularity of a time series on its corresponding coarse-graining scale^11,12^. More recently, heart rate complexity (HRC) shown as the area under the MSE curve were reported by Ho et al. (6–20 scales)^14^ and Tang et al.(1–20 scales)^15^. In specific clinical situations, MSE can exhibit marked changes where conventional HRV measurements do not^14,16^. Studies have also successfully used MSE to enhance the recognition of trauma patients at risk of poor outcomes^17,18^. Furthermore, HRC based on MSE analysis has also been reported useful in predicting outcomes in stroke^15,19^ and liver transplant^20^ patient outcomes. However, the utility of HRC based on MSE analysis in patients with end-stage liver disease has not been established and it warrants further study.

Despite a potential risk of cardiac failure and decompensation in the immediate and short term post-transplantation period, liver transplantation is currently the only method for reversing cirrhotic cardiomyopathy^3^. Carey et al. reported the QT interval prolongation improved 4 months after liver transplantation^21^. Luigi et al. 2010 reported that the standard deviation of normal-to-normal interval (SDNN) progressively increased after liver transplantation and was significantly higher at 12 and 33 months^22^. Salatini et al. reported a global HRV improvement 2 months after liver transplant^23^. However, no study has investigated the time course of the recovery of cardiac function in end-stage liver disease after liver transplantation.

The aims of our study were (1) to detect whether HRC based on MSE can differentiate the cardiac electrophysiological abnormalities based on the severity of end-stage liver disease and (2) to explore the time course of the reversal of HRC following liver transplantation.

## Materials and methods

### Patients

The present study was approved by the Research Ethics Committee of National Taiwan University Hospital, Taipei, Taiwan (200809046R) and was registered at http://clinicaltrials.gov with the identifier NCT00778687. After receiving Institutional Review Board approval of our study protocol and written informed consents from all participating patients, we consecutively enrolled 30 adult recipients aged 18–70 years old with end-stage liver disease who received living donor liver transplantation between October 2008 and January 2010. The exclusion criteria were as follows: below 20 years of age, history of pulmonary resection, chronic respiratory insufficiency, cardiac dysfunction (according to preoperative echocardiography), and failure of the surgery.

All relevant guidelines and regulations were followed by our institution. Living donor related liver transplants were recruited in this study. The recipients’ relatives donated portions of their livers. No allografts obtained from executed prisoners were used. Organ procurement for liver transplantation in this study was performed by the Division of Liver Transplantation, Department of Surgery, National Taiwan University Hospital. No information could lead to the identification of a patient in this study.

### Data acquisition

Electrocardiogram (ECG) recordings (recorded after 10 min of bed rest) were obtained from each patient for at least 30 min in the supine position at the following time points: (1) 1 day before surgery, (2) postoperative day (POD) 7, (3) POD 14, (4) POD 90, and (5) POD 180. A 30 min ECG recording at each time points is not a routine checkup, which need the patients complied with our protocol. We enrolled the participating patients after receiving written informed consents. Continuous ECG signals were recorded using a Holter monitor (MyECG E3-80, Microstar Company, Taipei, Taiwan); all signals were digitized and sampled at 1 kHz. Each heartbeat was annotated using an automatic arrhythmia detection algorithm, and each annotation was verified through visual inspection. R-R intervals are derived as the time intervals between two successive peaks of R-wave in EEG signals, which present interbeat intervals between all successive heartbeats. Normal-to-Normal (N–N) sinus interval are the intervals between normal heartbeats, in which artifacts have been removed.

### Assessment of heart rate variability

#### Analysis of multiscale entropy and heart rate complexity

The entropy measure of time series on time scale 1 was calculated using the original time series based the definition of sample entropy (SampEn). The entropy measures on time scales 2 ~ 20 were calculated as the SampEn values of the coarse-grained time series. Therefore, the MSE method incorporated two procedures:

1. The “coarse-graining” process
  For a given time series (with *N* data points noted as *x*_*1*_*, x*_*2*_*, x*_*3*_*, …, x*_*N*_), a coarse-grained time series was derived by averaging the data points with non-overlapping windows of coarse-graining time scale τ. Each element (noted as *y*_*j*_^*(τ)*^) of the coarse-grained time series can be calculated according to the following equation:
  1 $$y_{j}^{(\tau )} ) = \tfrac{1}{\tau }\Sigma_{i = (j - 1)\tau + 1}^{j\tau } x_{i}$$
  In which, $1\le {\text{j}}\le {\text{N}}/\uptau$. The data length of each coarse-grained time series is *N/τ.*
2. Calculation of sampEn
  SampEn was defined to reflect the probability that sequences that match each other for the first *m* data points would also match for the next point. There were two parameters of pattern length *m* and the tolerance *r* in the calculation of SampEn. Here, pattern length* m* is 2 and tolerance *r* is 20% of the sample deviation of time series. The calculation of SampEn was made as follows: For a time series with N data points forms *N-m* + *1* vectors *v*_*m*_*(i)* for $1\le i\le N-m+1$, where vector *v*_*m*_*(i)* = {*x*_*i*+k_: *0 ≦ k ≦ m-1}.* The distance between two vectors derived from time series was defined to be *d[v*_*m*_*(i), v*_*m*_*(j)]* = *max{|x*_*i*+*k*_*–x*_*j*+*k*_*|;0 ≦ k ≦ m-1}*. A match of two vectors was defined as *d[v*_*m*_*(i), v*_*m*_*(j)]* < *r.* For each selected template *v*_*m*_*(i)*, a template match *C*_*i*_^*m(r)*^ represented the probability that any vector *v*_*m*_*(j)* match *v*_*m*_*(i)* and *B*_*i*_^*m*^*(r)* represented the count of matches.* B*^*m*^*(r)* is the average amount of *B*_*i*_^*m*^*(r)*. Then, SampEn was obtained using the following equation:
  2 $${\text{SampEn}}(N,m,r) = - {\text{In}}\left[ {\frac{{\left( {N - m - 1} \right)^{ - 1} \Sigma_{i = 1}^{N - m - 1} B_{i}^{m + 1} \left( r \right)}}{{\left( {N - m} \right)^{ - 1} \Sigma_{i = 1}^{N - m} B_{i}^{m} \left( r \right)}}} \right]$$

In this study, we coarse-grained the original time series SampEn up to a scale factor of 20^11^.

The value of heart rate complexity was defined as the sum of the entropies of scales from 1 to 20 in this study^15,20^.

### Analysis of conventional heart rate variability

#### Time-domain factors of heart rate variability

The time-domain parameters of HRV included the following:

1. SDNN: SDNN reflects all the cyclic components responsible for variability during the recording period.
2. Root mean square of the differences between adjacent NN intervals (RMSSD).
3. Proportion of paired successive NN intervals that differ by more than 50 ms divided by the total number of NN intervals (pNN50).
4. Proportion of paired successive NN intervals that differ by more than 20 ms divided by the total number of NN intervals (pNN20).

#### Frequency-domain factors of heart rate variability

1. The low-frequency/high-frequency (LF/HF) ratio: LF/HF is associated with sympathetic/parasympathetic balance^24^.

### Statistical analysis

Demographic data are presented as mean values and standard deviations (SD) for continuous variables. Pearson’s correlation was used to describe the relationship between MELD scores and HRC analysis results. The differences in the parameters of the three MELD groups at each time point were analyzed through analysis of variance (i.e., ANOVA) tests. A generalized linear mixed model was used to evaluate the group effect, time effect and interaction between the two factors in HRC trends among the three MELD groups. A *P* value of < 0.05 was regarded as statistically significant. All statistical analyses were performed using SAS version 9.4 (SAS Institute, Cary, NC, USA).

## Results

### Patient baseline characteristics

Thirty liver transplant recipients (22 male and eight female patients) with a mean age of 54.1 ± 9.8 years were enrolled into the present study. The ECG recordings from the recipients were retrospectively stratified into three groups according to liver disease severity as assessed using model for end-stage liver disease (MELD) scores^25^: (1) MELD score of ≤ 10; (2) MELD score between 11 and 24; (3) MELD score of ≥ 25^26^. The parameters (comprising baseline characteristics and measurements of HRV) of the three groups were compared.

**Figure 1:**
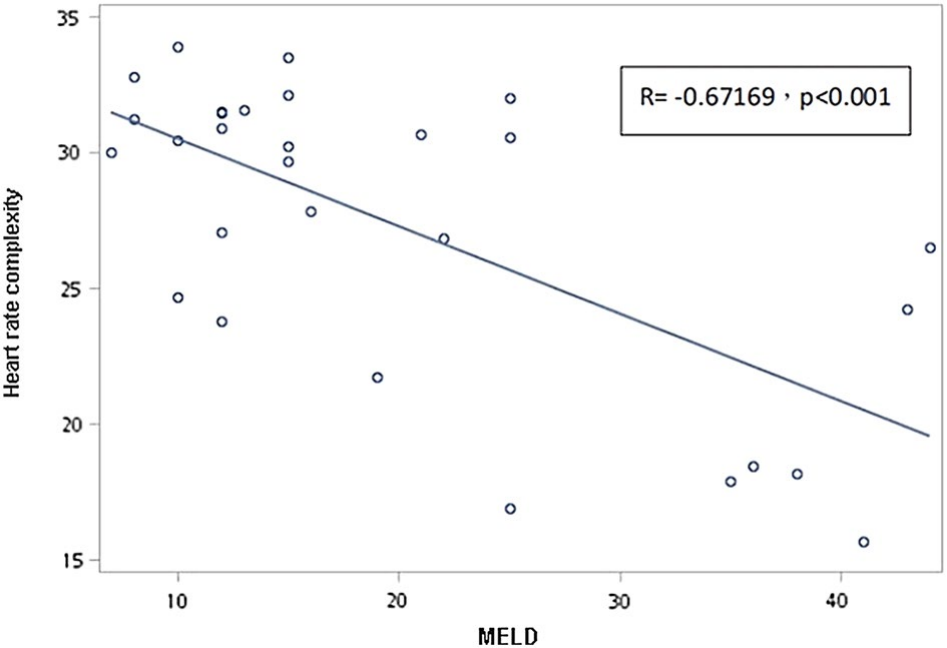
Correlation between model for end-stage liver disease (MELD) score and heart rate complexity.

Laboratory data and the factors derived from the heartbeat time series (HB-derived factors) were studied during the pre-transplantation stage. The patients’ characteristics are summarized in Table 1. Recipients with varying degrees of liver disease severity had a comparable average age, proportion of hypertension and diabetes mellitus, and similar β-blocker usage and albumin levels. There were no significant differences in revised cardiac risk index (RCRI)^27^, which is widely used to predict perioperative cardiac complications, among the three MELD groups. The patients’ creatinine, bilirubin, international normalized ratio (INR), and MELD scores were significantly different in analyses stratified by liver disease severity. Significant preoperative differences in HRC based on MSE analysis were observed among the three groups [30.6 (2.94), 29.1 (3.31), 21.2 (5.73)] (p < 0.001) while other HRV analyses that included SDNN (p = 0.2179), RMSSD (p = 0.8357), PNN50 (p = 0.2430), PNN20 (p = 0.1143) and HF/LF (p = 0.3371) showed no significant differences among the three groups. Furthermore, a significantly negative correlation was discovered between MELD score and HRC analysis result (Fig. 1).

**Table 1:** Baseline characteristics of three groups according to liver disease severity.

Parameters	MELD group 1	MELD group 2	MELD group 3	p-value
	N = 8(≦10 )	N = 14 (11–24)	N = 8 (≧25)	
Age (year)	52.9 (7.22)	54.4 (8.20)	51.1 (14.93)	0.7736
Gender				0.0766
Male	7	9	6	
Female	1	5	2	
Height	164.56(4.51)	165.56(8.24)	163.86(9.70)	0.8810
Weight	72.49(13.62)	65.54(13.12)	62.86(11.28)	0.3220
BMI	26.70(4.49)	23.86(4.17)	23.65(5.28)	0.3500
MELD score	9.1 (1.64)	16.0 (4.19)	35.9 (7.41)	< 0.0001
Hypertension	1	2	2	0.8365
DM	1	4	1	0.6052
RCRI	1.50 (0.756)	1.07 (0.267)	1.63 (0.744)	0.0749
β-blocker	1	2	1	1.0000
Total bilirubin (mg/dL)	1.46 (0.449)	5.64 (6.92)	29.45 (13.91)	< 0.0001
INR	1.08 (0.073)	1.42 (0.365)	2.05 (0.665)	0.0003
Creatinine (mg/dL)	0.98 (0.128)	0.96 (0.377)	2.19 (1.396)	0.0025
Albumin (g/dL)	3.92 (0.538)	3.45 (0.635)	3.40 (0.650)	0.1838
Heart beat derived				
HRC	30.6 (2.94)	29.1 (3.31)	21.2 (5.73)	< 0.0001
SDNN	60.0 (6.96)	53.5 (20.08)	43.8 (19.34)	0.2179
RMSSD	34.6 (5.36)	34.7 (13.16)	31.8 (11.21)	0.8357
PNN50	0.0636 (0.0436)	0.0628 (0.0810)	0.0184 (0.0234)	0.2430
PNN20	0.329 (0.1531)	0.234 (0.1988)	0.137 (0.1262)	0.1143
LF/HF	2.51 (1.181)	1.96 (1.384)	1.51 (1.1789)	0.3371

Values are presented as mean (standard deviation, SD).

*MELD* Model for end-stage liver disease, *DM* Diabetes mellitus, *RCRI* Revised cardiac risk index, *PT/INR* Prothrombin time/international normalized ratio, *HRC* Heart rate complexity, *SDNN* Standard deviation of normal-to-normal (NN) intervals, RMSSD Root mean square of successive differences, *PNN50* Proportion of paired successive *NN* intervals that differ by more than 50 ms divided by the total number of NN intervals, *PNN20* Proportion of paired successive NN intervals that differ by more than 20 ms divided by the total number of NN intervals, *LF/HF* A ratio of low frequency to high frequency.

**Figure 2:**
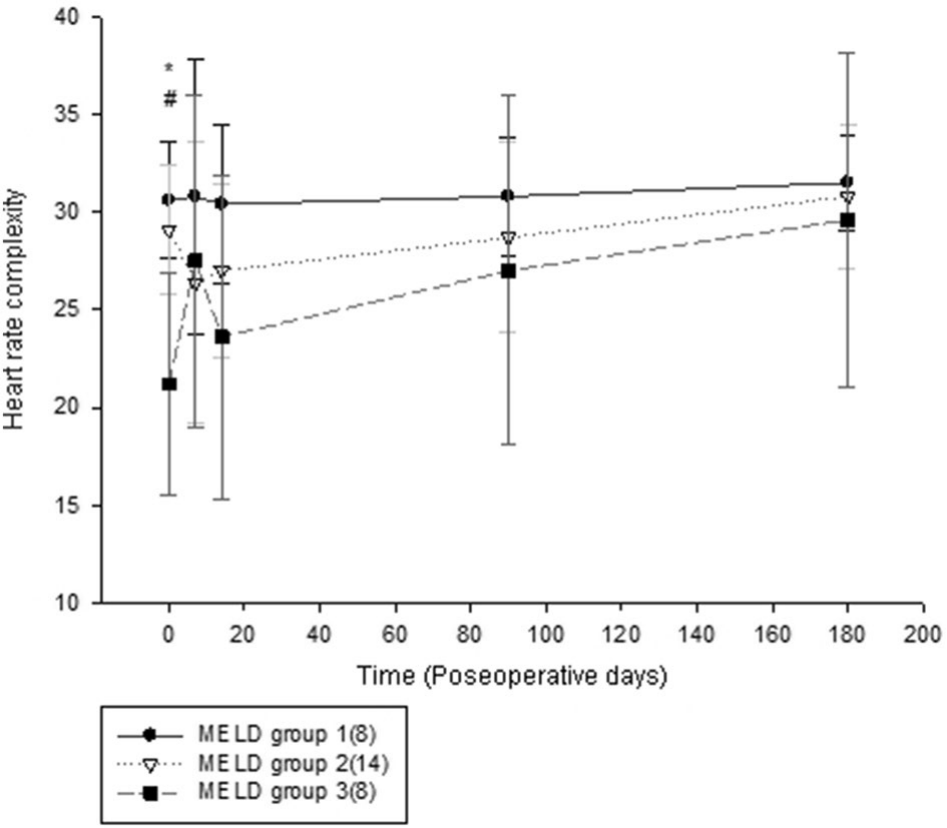
Heart rate complexity changes over time in three model for end-stage liver disease (MELD) groups.

**Figure 3:**
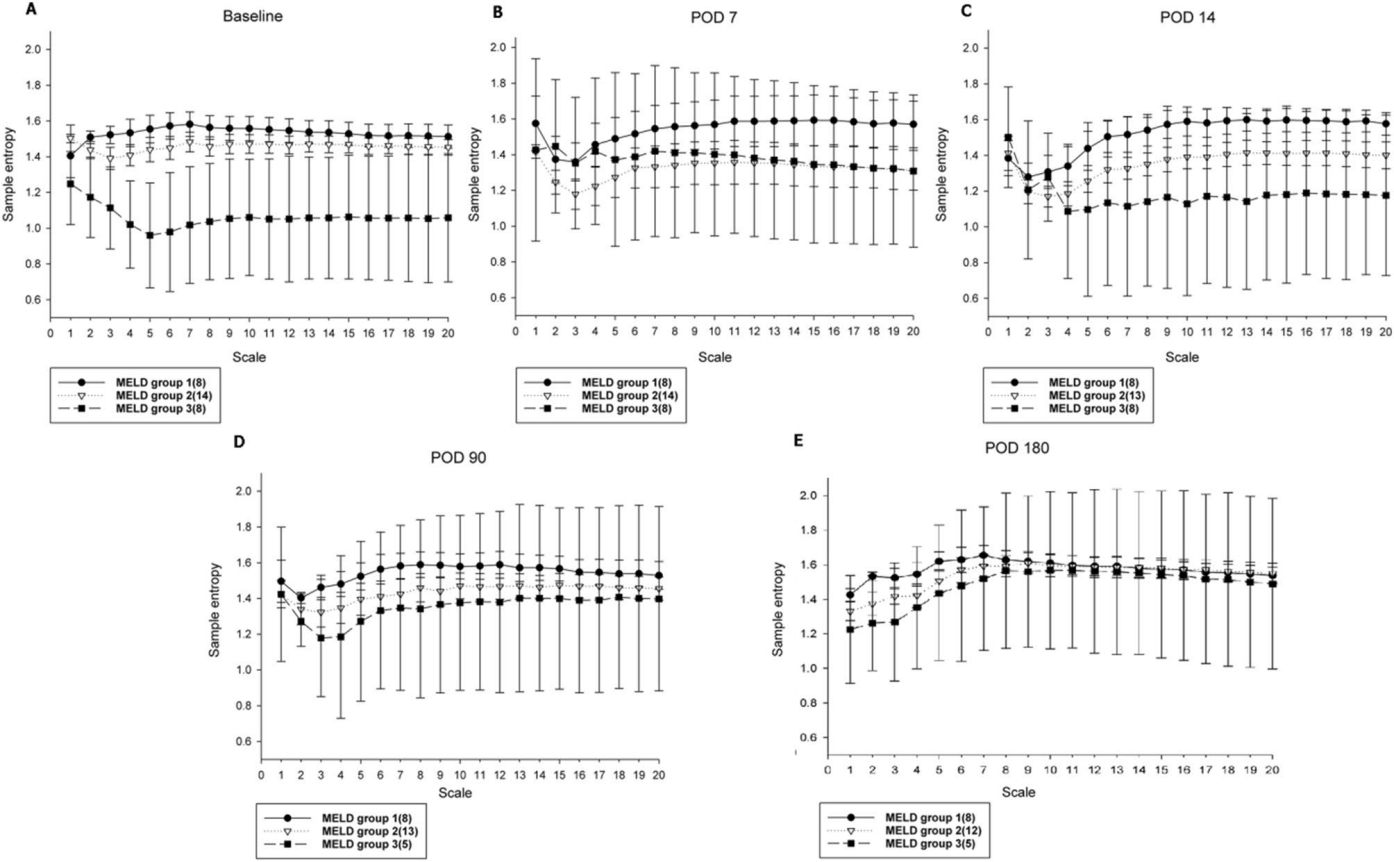
Multiscale entropy changes among three liver disease severity groups as assessed using model for end-stage liver disease (MELD) scores from (**A**) 1 day before surgery (Baseline), (**B**) postoperative day (POD) 7, (**C**) POD 14, (**D**) POD 90, and (**E**) POD 180. Group 1, MELD score of ≤ 10; Group 2, MELD score between 11 and 24; Group 3, MELD score of ≥ 25. Multiscale entropy calculates sample entropy (y axis) for different scales 1–20 (x axis). The original MSE curves in Group 3 were lowered in 1–20 scales compared to Group 1 and Group 2 before surgery. MSE in Group 3 increased following liver transplantation. Notably, they become comparable at larger scale factors among the three groups, particularly evident 7 days after liver transplantation.

While the three groups stratified by MELD score exhibited significant differences in HRC analysis before undergoing liver transplantation. HRC analysis revealed no significant difference among the three groups by POD 7 following liver transplantation and subsequent analyses at POD 90 and 180 continued to show no statistically significant difference, with a trend for HRC analysis result in Group 3 to further approximate the results in Groups 1 and 2 (Table 2). The generalized linear model showed that the preoperative HRC results were different between Group 3 vs Groups 1 and 2 combined, whereas no difference was identified between Groups 1 and 2 (group 3 vs group 1, p = 0.0002; group 3 vs group 2, p = 0.0015; group 2 vs group 1, p = 0.1975). The linear mixed model also revealed that the trend was significantly different in Group 3 compared to Group 1 and 2 (Fig. 2). (time group interaction: group 3 vs group 1, P = 0.0216; group 3 vs group 2, p = 0.0348; group 2 vs group 1, p = 0.5978). Similarly, the original MSE curves in Group 3 differed from Group 1 and Group2 before surgery. The sample entropies were apparently lower in group 3. They become comparable at larger scale factors over time among the three groups, which was particularly as soon as 7 days after liver transplantation. (Fig. 3).

**Table 2:** Changes of heart rate complexity during the period of investigation.

MELD (n)	Baseline	POD7	POD14	POD90	POD180
Group1(8)	30.6 (2.94)	30.8 (7.07)	30.4 (4.08)	30.8 (3.00)	31.5 (2.45)
Group2(14)	29.1 (3.31)	26.4 (7.18)	27.0(4.40)	28.7 (4.87)	30.8 (3.70)
Group3(8)	21.2 (5.73)	27.5 (8.47)	23.6(8.31)	27.0 (8.93)	29.6 (8.54)
ANOVA	0.0001	0.4363	0.1	0.4901	0.7951
p-value					

Values are presented as mean (standard deviation, SD).

*MELD* Model for end-stage liver disease, *POD* postoperative day, *ANOVA* analysis of variance.

Five patients died from different causes during the investigation period (two and three patients from MELD Group 2 and MELD Group 3, respectively). Among the five patients who died during the investigation period of the present study, one patient in MELD Group 2 developed acute heart failure combined with pulmonary hypertension during the ICU stay and died of arrhythmia on POD 46. No other cardiovascular events (including coronary artery disease and new-onset arrhythmia) were noted in the other 29 patients. Another patient in MELD Group 3 died of esophageal varices rupture with massive hemorrhage on POD 7. The other three patients died of uncontrolled infection with sepsis on POD 15, 57, and 155 respectively.

## Discussion

The main findings of the present study were as follow. (1) HRC analysis can be used to identify cardiac electrophysiological alteration in liver transplant patients according to liver disease severity. (2) A significantly negative correlation exists between HRC and MELD scores. (3) Improvement in end-stage liver disease-associated cardiac electrophysiological alterations detectable by HRC based on MSE can be observed following liver transplantation as early as POD 7.

Liver cirrhosis is frequently associated with autonomic dysfunction. The effects of autonomic nervous system modulation on cardiovascular signals is believed to be nonlinear physiologically^11,28^; however, most studies applied conventional linear models to analyze HRV. Tseng et al. verified that nonlinear MSE analysis is useful for evaluating autonomic nervous system through a table tilt test^29^. The present study is the first report on the application of HRC based on MSE for investigating the relationship between HRC and of liver disease severity. In our study, no significant difference identified in MELD Group 1 and 2. However, a marked decrease in HRC was noted in patients with MELD scores of ≥ 25. By contrast, although a decreasing trend was observed with respect to conventional HRV analysis (comprising time domain and frequency domain) that were conducted according to the severity of liver disease, no significant difference was identified. According to the results of the present study, HRC based on nonlinear MSE may provide greater sensitivity for evaluating electrophysiological abnormalities or autonomic dysfunction in the setting of end-stage liver disease.

The relationship between liver disease severity and the degree of autonomic dysfunction has been both supported^6–9^ and disputed^22,30^ by studies that employed different detection methods. Our results indicated a significantly negative correlation between HRC and liver disease severity. However, this correlation was less and insignificant using conventional HRV analysis. HRC was defined as the summation of entropies of scales from 1 to 20 in this study. It is possible that the complexity of HRV at the longer time scale more closely reflects certain behavior of systemic hemodynamic turbulences. Our results showed that HRC based on MSE (1–20 scales) is superior to conventional linear analyses of HRV, which is also compatible to previous findings by Chen et al. in stroke patients^19^.

Cirrhotic cardiomyopathy represents a significant management challenge in patients with end-stage liver disease. Liver transplantation is the only strategy for reversing the symptoms of end-stage liver disease, and the timing of cardiac recovery after liver transplantation varies substantially according to the evaluation methods and period of investigation. Carey et al. reported that most end-stage liver disease patients with prolonged QTc experience a significant improvement in 4 months after liver transplantation^21^. Torregrosa et al. showed that liver transplantation reversed cardiac alterations (related to ventricular wall thickness and diastolic dysfunction) in cirrhosis patients within 6 to 12 months following liver transplantation^31^. Fukazawa et al.^32^ prevailed that the reversal of cirrhotic cardiomyopathy could begin within one hour after reperfusion by evaluating the transmitral E/A ratio^32^. Barrata et al. 2010 reported that the decreased HRV partially improved after liver transplantation by measuring SDNN, however, their RMSSD and LF/HF measurements indicated no beneficial effects on parasympathetic impairment^22^. Salatini et al. reported a global HRV improvement 2 months after liver transplant^23^. The present study is the first study to prospectively evaluate the time course of recovery of cardiac complexity after liver transplantation. We prospectively collected HRV parameters including HRC based on MSE and conventional HRV analysis at the following time points: (1) 1 day before surgery; (2) postoperative day (POD) 7; (3) POD 14; (4) POD 90; and (5) POD 180. Although the investigation period was not the longest compared to previous studies, we found that HRC in patients with high MELD score could show improvement as early as 7 days after liver transplantation and became comparable even after 180 days.

The absence of rhythm variability is a poor prognostic factor in numerous situations^17,18,33^. Our results revealed that pretransplant HRC was significantly lower in the deceased patients relative to the surviving patients for 1-year mortality. This finding is compatible with those of other studies; that is, decreased HRV is associated with poor outcomes in cirrhotic patients^34,35^. Based on our results, we showed HRC may serve as a useful method for evaluating autonomic cardiac function before and after liver transplantation.

Our results showed a meaningful discovery that HRC based on MSE and the original MSE curves were significantly lowered in group 3 (MELD score of ≥ 25) before surgery. HRC and the original MSE curves in patients with MELD score ≧ 25 improved over time and became comparable to those with MELD < 25 as early as in 7 days. Although the sample size was limited, we still revealed compelling trends indicating the restoration of cardiac autonomic function after liver transplantation through the application of multiscale entropy analyses.

### Limitation

The major limitation of the present study is that the case number was limited. A follow-up study that include a larger study cohort size is needed to validate our findings and further delineate the potential clinical applications. Furthermore, other measures of cardiac function (e.g., diastolic dysfunction and pro-BNP levels) were not assessed in our study, the incorporation of other cardiac studies may further clarify the clinical utility of HRC analysis.

## Conclusion

Cardiac electrophysiological alterations in end-stage liver disease can be assessed through HRC based on MSE, which appears to provide improved sensitivity for evaluating autonomic dysfunction in end-stage liver disease than conventional linear HRV analysis. HRC in patients with MELD score ≥ 25 can show improvement as early as 7 days post liver transplantation.

## Data Availability

Data supporting the findings of this study are available from the corresponding author on request.

## References

[1] Wong F (2009). Cirrhotic cardiomyopathy. Hepatol. Int..

[2] Izzy M (2020). Redefining cirrhotic cardiomyopathy for the modern era. Hepatology.

[3] Lee RF, Glenn TK, Lee SS (2007). Cardiac dysfunction in cirrhosis. Best Pract. Res. Clin. Gastroenterol..

[4] Stein PK, Bosner MS, Kleiger RE, Conger BM (1994). Heart rate variability: A measure of cardiac autonomic tone. Am. Heart J..

[5] La Rovere MT (2003). Short-term heart rate variability strongly predicts sudden cardiac death in chronic heart failure patients. Circulation.

[6] Mohamed R, Forsey PR, Davies MK, Neuberger JM (1996). Effect of liver transplantation on QT interval prolongation and autonomic dysfunction in end-stage liver disease. Hepatology.

[7] Genovesi S (2009). QT interval prolongation and decreased heart rate variability in cirrhotic patients: relevance of hepatic venous pressure gradient and serum calcium. Clin. Sci..

[8] Ates F (2006). The relationship of heart rate variability with severity and prognosis of cirrhosis. Dig. Dis. Sci..

[9] Fleisher LA, Fleckenstein JF, Frank SM, Thuluvath PJ (2000). Heart rate variability as a predictor of autonomic dysfunction in patients awaiting liver transplantation. Dig. Dis. Sci..

[10] Richman JS, Moorman JR (2000). Physiological time-series analysis using approximate entropy and sample entropy. Am. J. Physiol.-Heart C.

[11] Costa M, Goldberger AL, Peng CK (2002). Multiscale entropy analysis of complex physiologic time series. Phys. Rev. Lett..

[12] Costa M, Goldberger AL, Peng CK (2005). Multiscale entropy analysis of biological signals. Phys. Rev. E Stat. Nonlinear Soft Matter Phys..

[13] Peng CK, Costa M, Goldberger AL (2009). Adaptive data analysis of complex fluctuations in physiologic time series. Adv. Adapt. Data Anal..

[14] Ho YL, Lin C, Lin YH, Lo MT (2011). The prognostic value of non-linear analysis of heart rate variability in patients with congestive heart failure–a pilot study of multiscale entropy. PloS ONE.

[15] Tang SC (2015). Complexity of heart rate variability predicts outcome in intensive care unit admitted patients with acute stroke. J. Neurol. Neurosurg. Psychiatry.

[16] Herlitz GN (2015). Physiologic variability at the verge of systemic inflammation: Multiscale entropy of heart rate variability is affected by very low doses of endotoxin. Shock.

[17] Riordan WP, Norris PR, Jenkins JM, Morris JA (2009). Early loss of heart rate complexity predicts mortality regardless of mechanism, anatomic location, or severity of injury in 2178 trauma patients. J. Surg. Res..

[18] Norris PR, Anderson SM, Jenkins JM, Williams AE, Morris JA (2008). Heart rate multiscale entropy at three hours predicts hospital mortality in 3,154 trauma patients. Shock.

[19] Chen CH (2015). Complexity of heart rate variability can predict stroke-in-evolution in acute ischemic stroke patients. Sci. Rep..

[20] Chan KC, Yeh JR, Sun WZ (2017). The role of autonomic dysfunction in predicting 1-year mortality after liver transplantation. Liver Int..

[21] Carey EJ, Douglas DD (2005). Effects of orthotopic liver transplantation on the corrected QT interval in patients with end-stage liver disease. Dig. Dis. Sci..

[22] Baratta L (2010). Long-term effect of liver transplantation on cirrhotic autonomic cardiac dysfunction. Dig. Liver Dis..

[23] Salatini R (2022). Cardiac autonomic modulation in children with severe liver disease, before and after liver transplantation. Transl. Pediatr..

[24] Malik, M. *et al. *Heart rate variability. Standards of measurement, physiological interpretation, and clinical use. Task Force of the European Society of Cardiology and the North American Society of Pacing and Electrophysiology. *Eur. Heart J.* **17**, 354–381 (1996). 8737210

[25] Kamath PS (2001). A model to predict survival in patients with end-stage liver disease. Hepatology.

[26] Guan R, Lui HF (2011). Treatment of hepatitis B in decompensated liver cirrhosis. Int. J. Hepatol..

[27] Ford MK, Beattie WS, Wijeysundera DN (2010). Systematic review: Prediction of perioperative cardiac complications and mortality by the revised cardiac risk index. Ann. Intern. Med..

[28] Manor B (2010). Physiological complexity and system adaptability: Evidence from postural control dynamics of older adults. J. Appl. Physiol..

[29] Tseng L (2013). Nonlinear and conventional biosignal analyses applied to tilt table test for evaluating autonomic nervous system and autoregulation. Open biomed. Eng. J..

[30] Oliver MI (1997). Autonomic dysfunction in patients with non-alcoholic chronic liver disease. J. Hepatol..

[31] Torregrosa M (2005). Cardiac alterations in cirrhosis: reversibility after liver transplantation. J. Hepatol..

[32] Fukazawa K (2009). Is the immediate reversal of diastolic dysfunction of cirrhotic cardiomyopathy after liver transplantation a sign of the metabolic etiology?. Liver transpl..

[33] Cheng D, Tsai SJ, Hong CJ, Yang AC (2009). Reduced physiological complexity in robust elderly adults with the APOE epsilon4 allele. PloS ONE.

[34] Oyelade T (2020). Heart rate turbulence predicts survival independently from severity of liver dysfunction in patients with cirrhosis. Front. Physiol..

[35] Oyelade T (2021). Heart rate variability in patients with cirrhosis: A systematic review and meta-analysis. Phys. Meas..

